# Hard and Highly Adhesive AlMgB_14_ Coatings RF Sputtered on Tungsten Carbide and High-Speed Steel

**DOI:** 10.3390/ma16216930

**Published:** 2023-10-28

**Authors:** Alexander M. Grishin, Vadim V. Putrolaynen

**Affiliations:** 1 Division of Electronics and Embedded Systems, School of Electrical Engineering and Computer Science, KTH Royal Institute of Technology, Kista, SE-164 40 Stockholm, Sweden; 2 INMATECH Intelligent Materials Technology, SE-127 51 Skärholmen, Sweden; 3 Department of Electronics and Electrical Power Engineering, Institute of Physics and Electronics, Petrozavodsk State University, 185000 Petrozavodsk, Russia

**Keywords:** micro and nanohardness, elastic strain index, resistance to plastic deformation ratio, elastic recovery ratio, friction coefficient, work of adhesion

## Abstract

We report a new industrial application of aluminum magnesium boride AlMgB_14_ (BAM) coatings to enhance the hardness of tungsten carbide ceramic (WC-Co) and high-speed steel tools. BAM films were deposited by RF magnetron sputtering of a single dense stoichiometric ceramic target onto commercial WC-Co turning inserts and R6M5 steel drill bits. High target sputtering power and sufficiently short target-to-substrate distance were found to be critical processing conditions. Very smooth (6.6 nm RMS surface roughness onto Si wafers) and hard AlMgB_14_ coatings enhance the hardness of WC-Co inserts and high-speed R6M5 steel by a factor of two and three, respectively. Complete coating spallation failure occurred at a scratch adhesion strength of 18 N. High work of adhesion and low friction coefficient, estimated for BAM onto drill bits, was as high as 64 J/m^2^ and as low as 0.07, respectively, more than twice the surpass characteristics of N-doped diamond-like carbon (DLC) films deposited onto nitride high-speed W6Mo5Cr4V2 steel.

## 1. Introduction

Such recent advances in machining technologies as high-speed, high-performance, and high-feed machining raise forced demands on the properties of coated tools regarding wear and oxidation resistance, surface lubricity, resistance to metal fatigue, and thermal shock. The extreme material properties of aluminum magnesium boride AlMgB_14_ respond to these challenge and promise a wide range of industrial applications. Exceptional hardness ranging from 45 to 51 GPa and low friction coefficients were demonstrated for the first time in 2003 in Ames Lab for pulsed-laser-deposited (PLD) thin AlMgB_14_ films [1]. Soon after, New Tech Ceramics Inc., based on the Ames Lab’s invention [2], started to commercialize aluminum magnesium boride and coined the name BAM for a rich family of materials that combine AlMgB_14_ with another hard boride, carbide, or boron nitride ceramics [3,4]. Despite a strong commercialization effort, PLD for a long time remained the only technique to obtain reliable characteristics of BAM coatings [5,6,7,8,9,10,11,12].

Fabrication of high-quality ultra-hard BAM films by RF magnetron sputtering of stoichiometric ceramic AlMgB_14_ was an important step in the exploration of AlMgB_14_. High target sputtering power and a short target-to-substrate distance enabled a high-energy ballistic impact regime of the adatoms’ motion that led to enhanced hardness and Young’s modulus by 25% and 60%, respectively, compared to PLD-made films. Stoichiometric in-depth compositionally homogeneous 2 μm thick films on a Si (100) wafer possess Vickers hardness peak values of 88 GPa and a Young’s modulus of 517 GPa at a penetration depth of 26 nm and, respectively, 35 GPa and 275 GPa at 200 nm in depth [13]. The friction coefficient was found to be 0.06, and the coating scratch adhesion strength was 14 N at the first chipping and 21 N at the film’s failure spallation yielded a work of adhesion as high as 18.4 J/m^2^ onto the Si wafer [14]. To achieve the best BAM film characteristics on curved surfaces of extended 3D objects we developed a two-step sputtering process. The first thin layer was deposited as a template at low RF power, facilitating layer-by-layer (Frank van der Merwe) mode growth of smooth films. The next layer is grown at high RF target sputtering power. The affinity of the subsequent flow of sputtered atoms to an already evenly condensed template fosters the development of a smooth surface of a hard BAM film [15].

The most recent advances and new results include the following: hard and lubricant BAM films produced by DC magnetron sputtering of three elemental targets (B, Mg, Al) [16], employing the process of RF plasma sputtering of AlMgB_14_ powder targets [17], AlMgB_14_/TiB_2_ composite ceramic brazed onto 304 stainless steel using a commercial AgCuTi eutectic foil [18], examination of BAM-coated twist drill wear, the evolution of cutting-edge geometry in drilling experiments with carbon-fiber-reinforced plastic (CFRP) laminate [19] and the novel application of BAM-based ceramic coatings onto the blade edges of razor blades [20] and onto the surfaces of rolling elements in bearing assemblies [21]. Especially noteworthy is the deep nanostructural characterization of the mechanical–tribological behavior and the analysis of the wear rate of nanocomposite TiSiCN coatings deposited on high-Co-based high-speed steel (ASSAB 17) burins [22].

In the present paper, we explore the properties of BAM films RF magnetron sputtered onto industrial cutting tools. BAM coatings enhanced the hardness of WC-Co inserts by a factor of two. Meanwhile, R6M5 steel drills became more than three times as hard after BAM coating with a friction coefficient twice as lowas the one acquired in the combined process of steel nitriding and N-doped diamond-like carbon coating [23].

## 2. Materials and Methods

**Figure 1:**
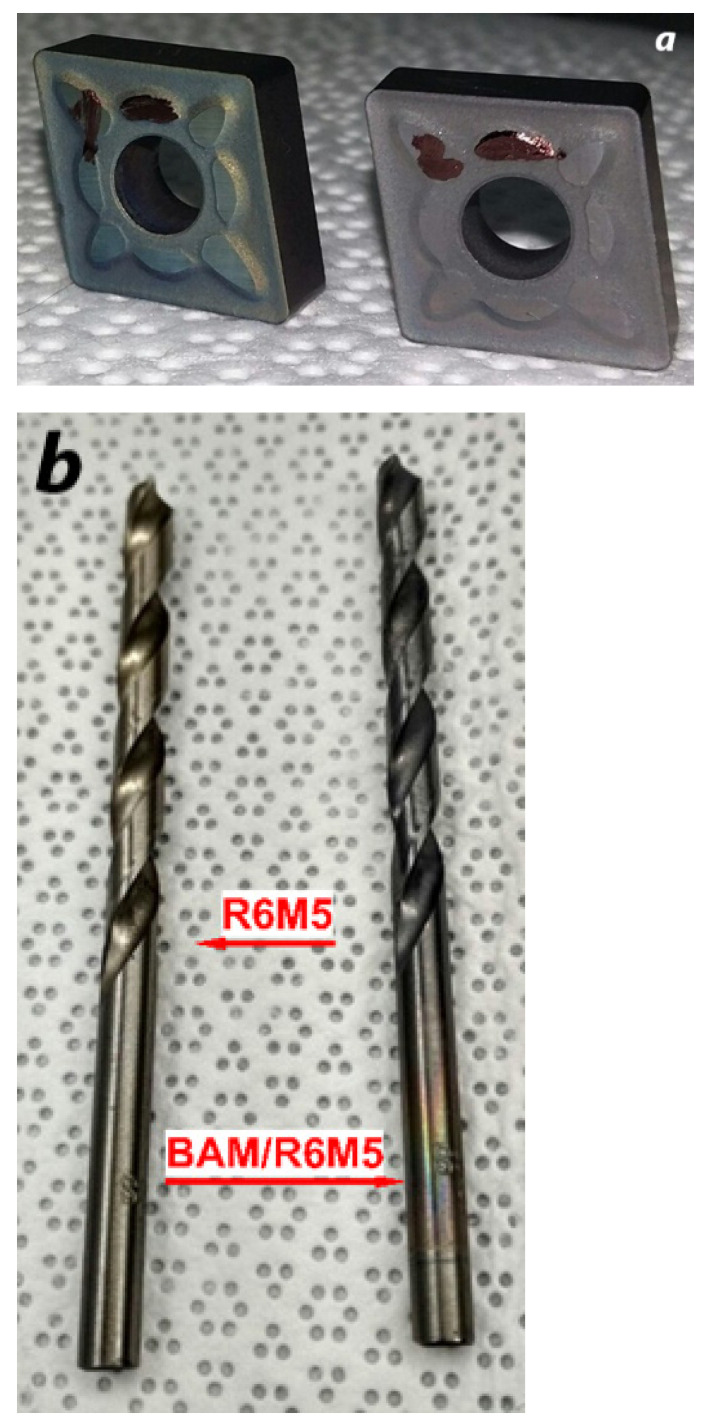
Photos of the commercial industrial tools used for the experiments. (**a**) Two WC-Co-based cemented carbide turning inserts processed with a BAM coating. Right is the BALINIT LATUMA^TM^ (AlTiN) specimen. (**b**) Untarnished and BAM-coated R6M5 drill bits.

Two turning inserts from WC-Co cemented carbides (composition classification K30 ISO 513) and R6M5 steel (W6Mo5Cr4V2, wt% [24]) drill bits, as shown in Figure 1, were used as substrates. One commercial turning insert was made using OERLIKON BALZERS’ surface-hardening technology with the BALINIT LATUMA^TM^ AlTiN coating (Norsborg, Sweden).

Hard aluminum magnesium boride (BAM) films were deposited by means of RF magnetron sputtering at processing conditions like the ones we published earlier [13,15]. In brief, the stoichiometric ceramic Al_0.75_Mg_0.78_B_14_ target (2.44 g/cm^3^, which is 94% of the theoretical density) was sputtered in an *AJA Orion* 5 vacuum chamber with an ultimate pressure 3 × 10^−7^ Torr. Plasma etching of the substrates with 15 min of target pre-sputtering preceded the deposition of BAM films, carried out at 4 mTorr of Ar gas pressure. A distance of 6.5 cm between the substrates and 200 W powered 2-inch magnetron (RF power density of 10 W/cm^2^) was found to be optimal to achieve hard, smooth, and highly adhesive BAM coatings at a deposition rate of 0.25 nm/s and a thickness of 3 μm.

High-resolution electron microscopy, X-ray, and electron diffraction proved that these films were amorphous (see ref. [25]). The chemical composition of the sputtered BAM films was analyzed earlier by EDS [15] and glow discharge optical emission spectroscopy (GDOES) [13]. The GDOES tool [26] ascertained the following processing conditions to achieve stoichiometric transfer compositions between the target and coatings: 15–25 cm short target-to-substrate distance, 10 W/cm^2^ RF sputtering power density, and a substrate temperature of 250–350 °C.

*NT-MDT INTEGRA Prima* (S/N 081503-16-001) atomic force microscope (AFM) proved that the BAM films’ roughness did not exceed the roughness of the trial glass substrates and Si wafers. Nanohardness indentation was accomplished using the *TTX-NHT2 CSM Instruments SA* (S/N 01-05821) with a Berkovich three-sided diamond pyramidal tip. Coatings adhesion tests (ASTM C 1624-05, ASTM D 7027-05) were performed using the *CSM Instruments SA Revetest^®^* (S/N 01-03079) tester equipped with a diamond *Rockwell C* indenter with a 200 μm radius under a high-resolution analytical *Tescan Mira_3_ LMU* scanning electron microscope (SEM) and optical control.

## 3. BAM-Coated WC-Co Inserts

### 3.1. Microstructure

**Figure 2:**
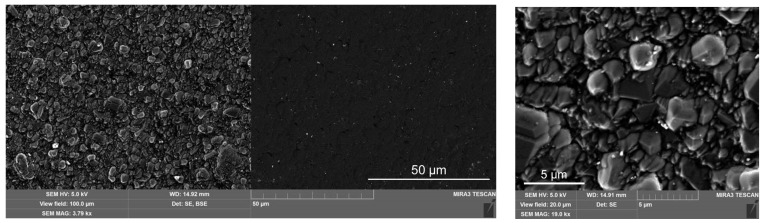

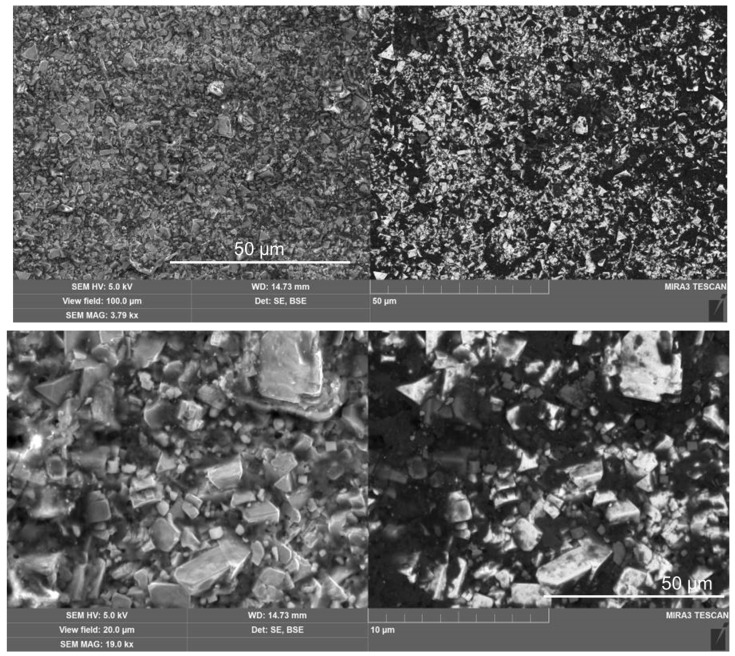
SEM micrographs showing the surface topography of the WC-Co inserts in the secondary electrons (SE) and back-scattered electrons (BSE) modes. Two upper frames—BAM-coated insert; two lower frames—the virgin surface of the uncoated insert.

**Figure 3:**
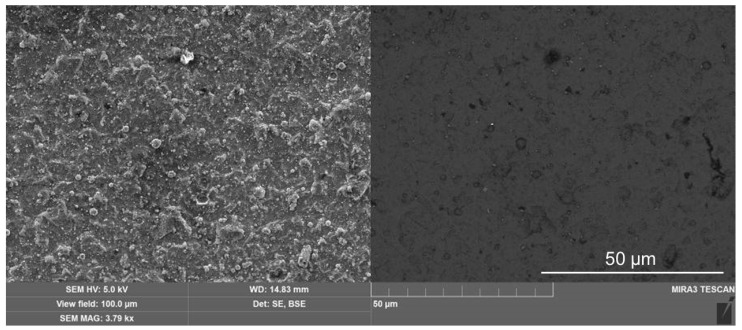

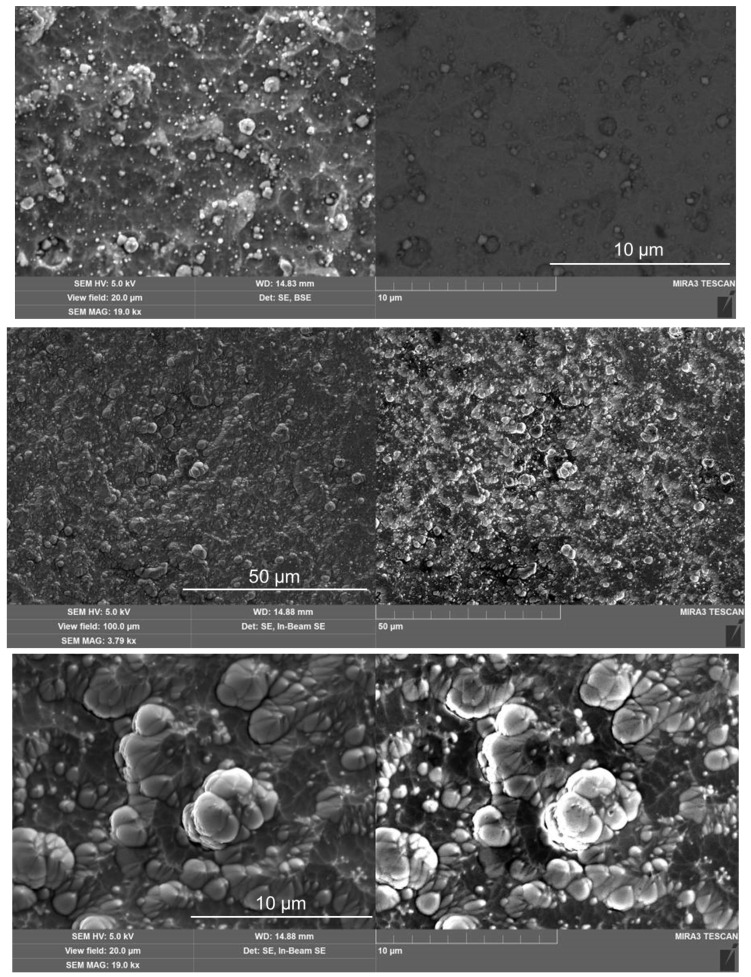
SEM images of the BALINIT LATUMA^TM^ WC-Co insert surfaces. Two upper frames—BAM-coated AlTiN/WC-Co insert; two lower frames—the virgin surface of the AlTiN/WC-Co insert.

Figure 2 and Figure 3 depict the surface morphology of the WC-Co inserts. Back-scattered SEM images make evident the amorphous nature of the uniform BAM coatings. Deposited BAM material evenly covers all the tungsten carbide submicron crystals. Figure 3 shows the presence of 1–2 μm microdroplets over the AlTiN surface in the BALINIT LATUMA^TM^ WC-Co specimen. The BAM film succeeds in screening not only all these droplets but also sparse micropores (micro holes) at the surface of the AlTiN coating.

### 3.2. Nanoindentation, Vickers Hardness

**Figure 4:**
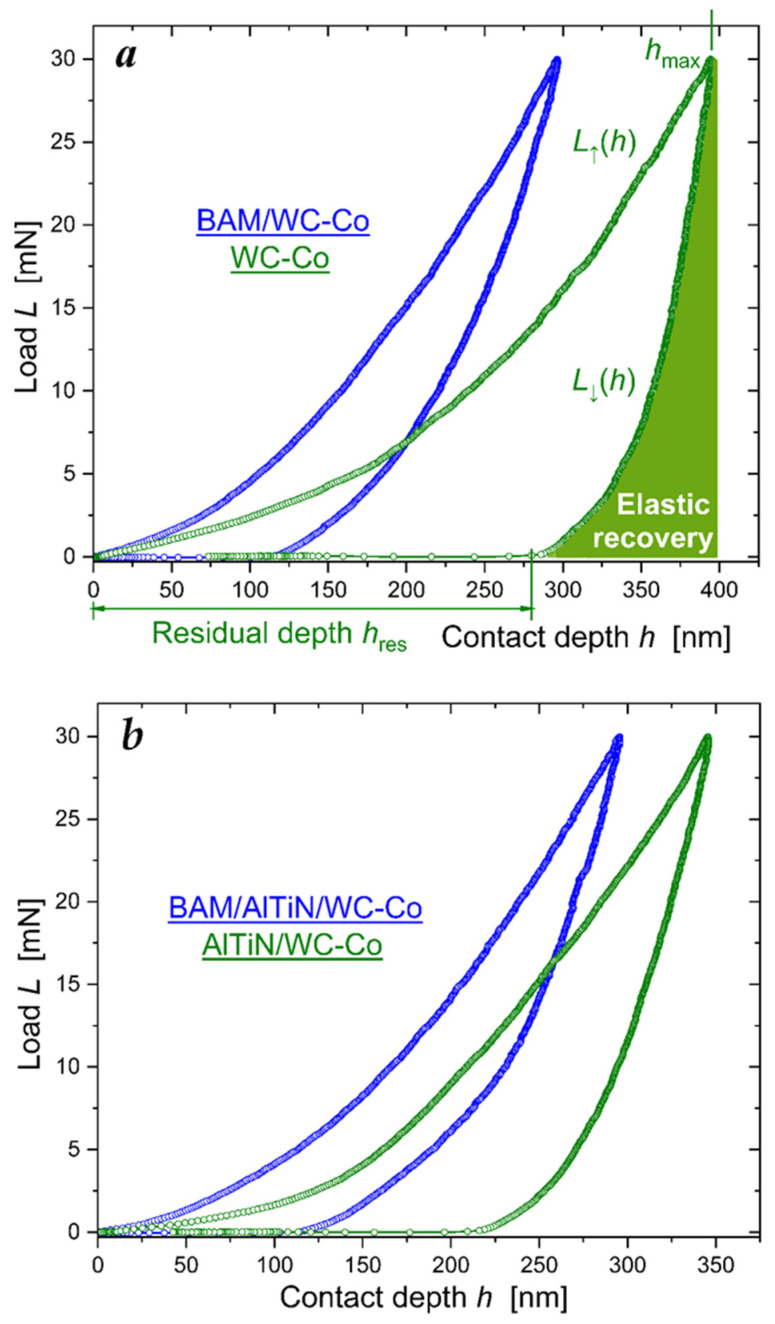
Load *L*—contact depth *h* curves: (**a**) in the WC-Co insert; (**b**) in the BALINIT LATUMA^TM^ (AlTiN) WC-Co insert. Blue symbols show the BAM-coated specimens, and green symbols show nanoindentation of the virgin surfaces of the WC-Co inserts.

The *TTX-NHT2 CSM Instruments SA* was employed to make nanoindentations in the coatings with a maximum load of 30 mN. At least five notches were made for each of the loads, with zero holding time and a total exposure time (for applying and removing the load) of 30 s. Figure 4 shows typical loading $L_{↑}\left(h\right)$ (ascending) and unloading $L_{↓}\left(h\right)$ (descending) versus contact depth *h* curves. The area between the $L_{↓}\left(h\right)$ and $L_{↓}\left(h\right)$ curves represents the energy dissipated in the material due to plastic deformation, whereas the area under the unloading curve $L_{↓}\left(h\right)$ defines the recovered work of elastic forces.

The loading–unloading *L*(*h*) curves were used to determine three main material characteristics of hardness *H*, effective Young’s modulus $E^{*}=E/\left(1-\nu^{2}\right)$, and elastic recovery ratio $R_{e}$. *H* and $E^{*}$ are calculated as per the Oliver and Pharr method fitting descending $L_{↓}\left(h\right)=A{\left(h-h_{res}\right)}^{m}$ dependence to the experimental unloading force curve [27]. We choose the Poisson’s coefficient ν to be equal to 0.25, 0.29, and 0.19 for BAM, AlTiN and tungsten carbide, respectively. The elastic recovery ratio is defined below as a ratio of areas under the unloading $L_{↓}\left(h\right)$ and loading $L_{↑}\left(h\right)$ curves:

(1) $$R_{e}=\int_{h_{res}}^{h_{max}}dh\cdot L_{↓}\left(h\right)/\int_{0}^{h_{max}}dh\cdot L_{↑}\left(h\right).$$

From the ascertained values of *H* and $E^{*}$ we calculated the dimensionless elastic strain index *H*/E* and the resistance to plastic deformation ratio *H*^3^/E*^2^, as presented in Table 1 and Table 2.

In addition, a Vickers microhardness HV0.2 was also measured using the microhardness tester ПMT-3 with a maximum load of 200 gf. The results are shown in Table 3.

Comparing these data, we conclude that BAM coating doubles the hardness of uncoated WC-Co inserts and increases the hardness of AlTiN/WC-Co by 1.6.

### 3.3. Adhesive Wear

**Figure 5:**
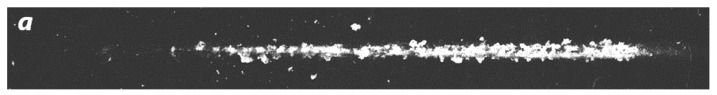

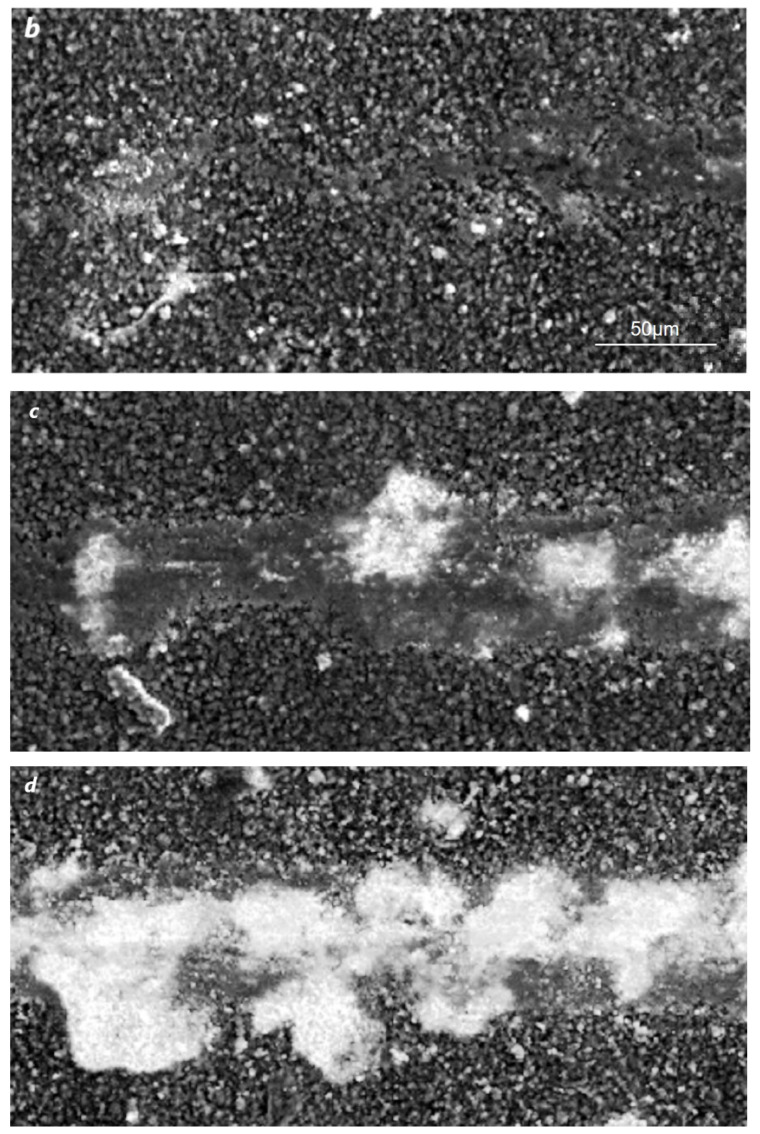
Progressive load scratch on the surface of the BAM-coated WC-Co insert. (**a**) General image of the 3 mm long scratch obtained over a loading range of 0–30 N. (**b**–**d**) Secondary electron SEM images of the initial scratch’s track segments at loading force values *L* = 0.9, 5 and 9 N, respectively.

**Figure 6:**
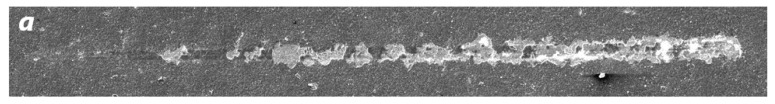

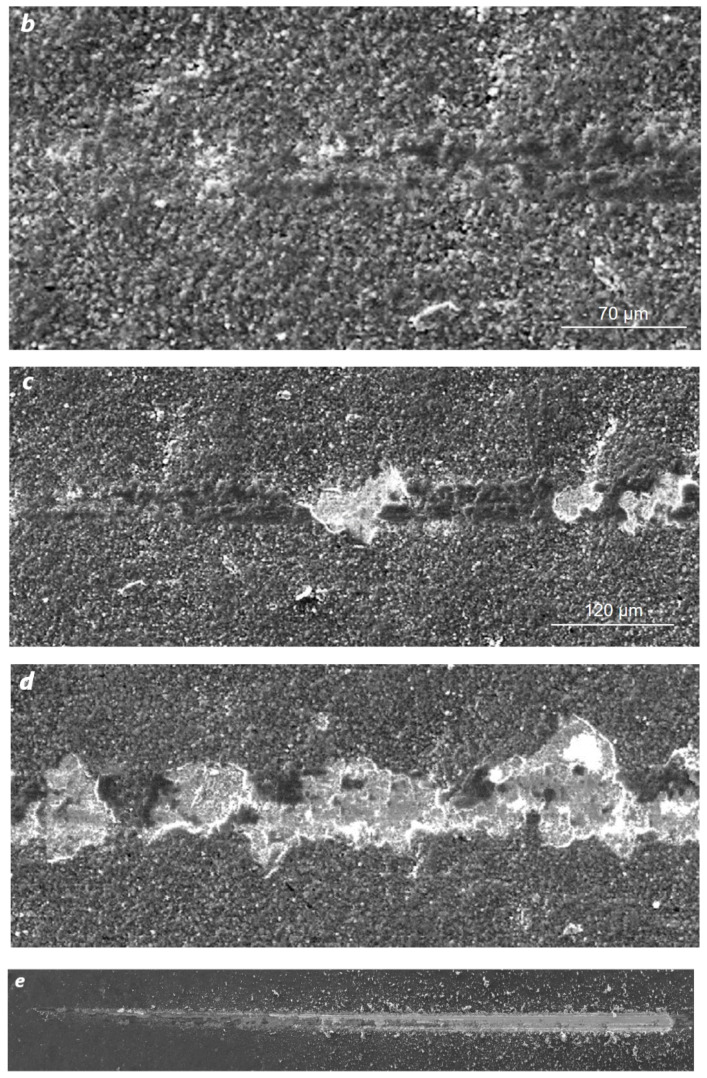
Progressive load scratch on the surface of the BAM-coated AlTiN/WC-Co insert. (**a**) General image of the 3 mm long scratch obtained over the loading range of 0–30 N. (**b**–**d**) Secondary electron SEM images of scratch segments at loading force values *L* = 1.5, 5 and 17 N, respectively. (**e**) Track of the indenter loaded from 1 to 61 N.

Evaluation of the abrasion properties of BAM coatings onto WC-Co and AlTiN/WC-Co inserts was performed according to the adhesion strength ASTM C1624-05(2015) and scratch resistance ASTM D7027-13 standards as follows. First, 3 mm long scratches were made by applying a linear progressive normal load *L* from 0.03 to 30 N with a rate of 30 N/min. Three critical load values *L*_c*i*_ (*i* = 1, 2, 3) were determined by inspecting the scratch tracks, both optically and from SEM images in Figure 5 and Figure 6, as well as examining distinctive marks in the loading curves of the displacement of the indenter *h*, tangential force, and acoustic emission intensity.

In acoustic emission signal, the first cracks in the BAM film onto WC-Co inserts are readily apparent at *L*_c1_ = 0.9 N. Peeling of the BAM coating from the tungsten carbide substrates at *L*_c2_ = 5 N becomes clear from the images of scratches obtained using the *Tescan Mira_3_ LMU* scanning electron microscope.

As for a scratch test for the BAM-coated BALINIT LATUMATM AlTiN/WC-Co specimen, the appearance of cracks can only be confidently judged by the signal from the acoustic emission sensor. On average, initiation of the first cracks occurs at *L*_c1_ = 1.7 Delamination of the BAM coating from the AlTiN/WC-Co substrates is clear from the SEM images of the scratches in Figure 6. On average, the peeling off of a single BAM layer from the AlTiN appears at *L*_c2_ = 5 N, while AlTiN peeling occurs at 12 N. Finally, complete destruction of the AlTiN layer happens at 41.

## 4. BAM-Coated R6M5 Steel Drill Bits

### 4.1. Hardness and Young’s Modulus

**Figure 7:**
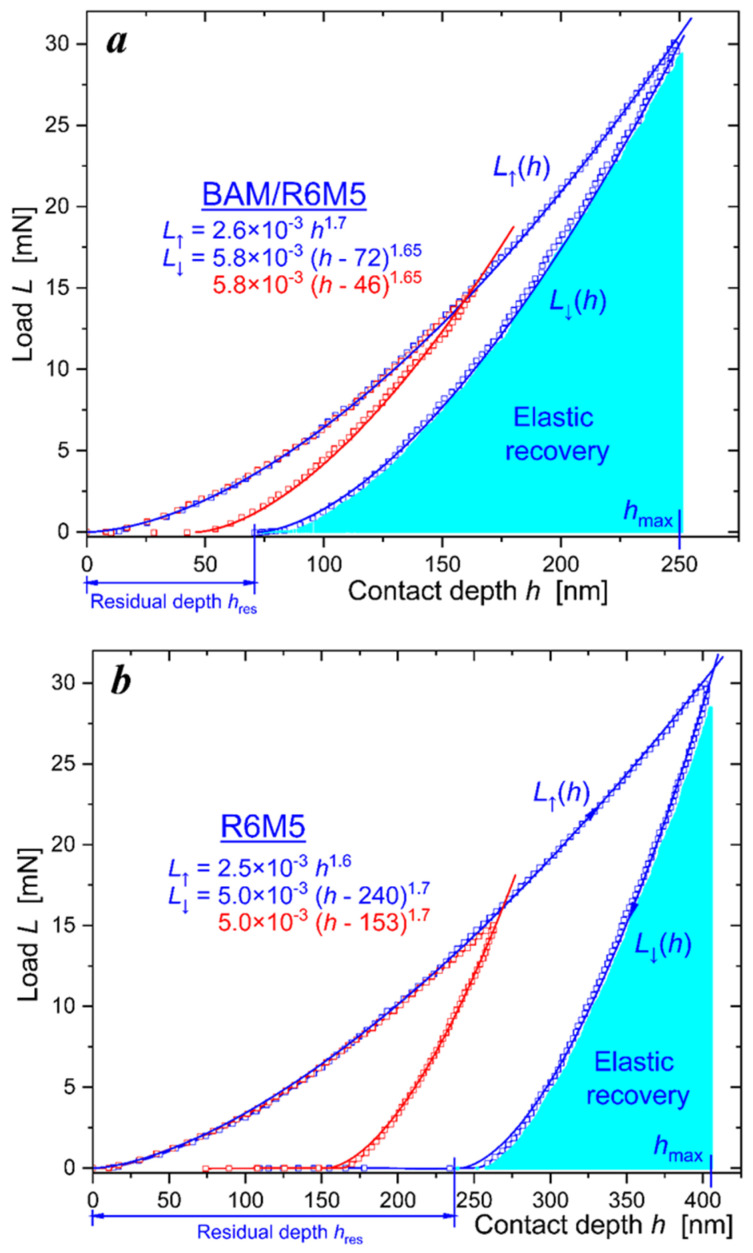
Load *L*—contact depth *h* curves: (**a**) BAM-coated R6M5 steel; (**b**) nanoindentation of the virgin surface of the R6M5 steel. Red and blue symbols depict two load-displacement curves shown in succession.

The loading–unloading curves in Figure 7 were used to calculate, as per the Oliver and Pharr method, the main mechanical properties of the BAM-coated R6M5 steel and compare them with those characterized in the uncoated R6M5 specimen. Poisson’s coefficient for the R6M5 steel was chosen to be equal 0.295. Data presented in Table 4 evidence the more than triple increase in hardness *H* and, as a consequence, twice as big recovery ratio *R*_e_ in the BAM-coated R6M5 drill bit.

Both diminished abrasive wear and high fracture toughness are still strong desirable characteristics for ceramic coatings. The experimental results, presented in Table 4, serve as indicative measures of the high abrasive resistance of AlMgB_14_-coated R6M5 steel. It is commonly assumed that a material with a high elastic strain index *H*/E*, like 0.1, possesses a better wear resistance than a material with a low ratio *H*/E*~0.01. The BAM/R6M5 steel drill exhibited a *H*/E* = 0.13, twice that of uncoated R6M5. Experimenting with a wide range of coating materials, J. Musil found the cooperative influence of parameter *H*/E* and the plastic deformation ratio *H*^3^/E*^2^ on the fracture toughness. The number of surface cracks notably decreased at higher *H*/E* and *H*^3^/E*^2^ values [28,29]. The parameter *H*^3^/E*^2^ was enhanced by a factor of ten after BAM coating of the R6M5 specimens. A concurrent increase in both parameters *H*/E* and *H*^3^/E*^2^ conforms to the general result J. Musil established for hard nanocomposite coatings [30].

### 4.2. Adhesive Strength

**Figure 8:**
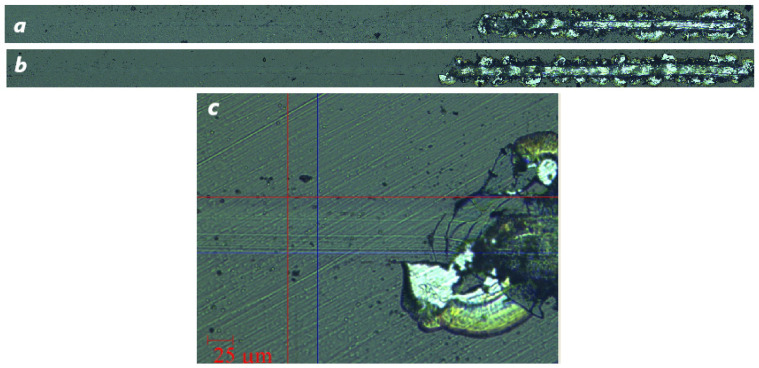
Progressive load scratch on the surface of BAM-coated R6M5 steel. (**a**,**b**) General image of two 5 mm long scratches obtained over a loading range *L* = 0–30 N. (**c**) Magnified optical image of a 400 μm long segment of scratch (**b**) at a loading force value *L*_c_ = 17 N.

Standard ASTM C 1624-05 and ASTM D 7027-13 scratch tests shown in Figure 8 revealed the very high adhesion properties of BAM films deposited onto high-speed R6M5 steel.

**Figure 9:**
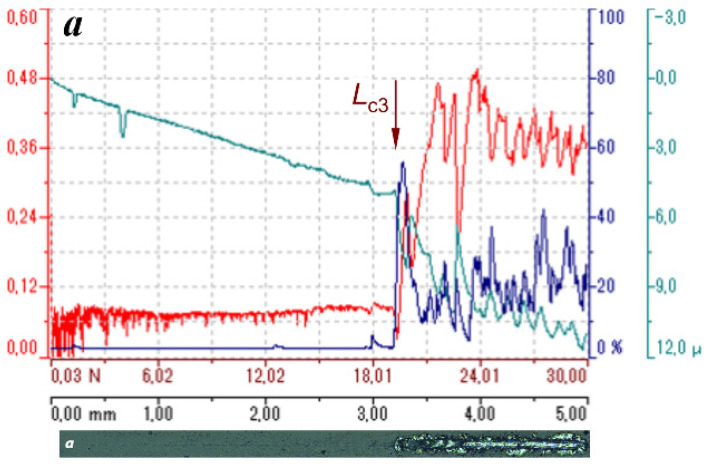

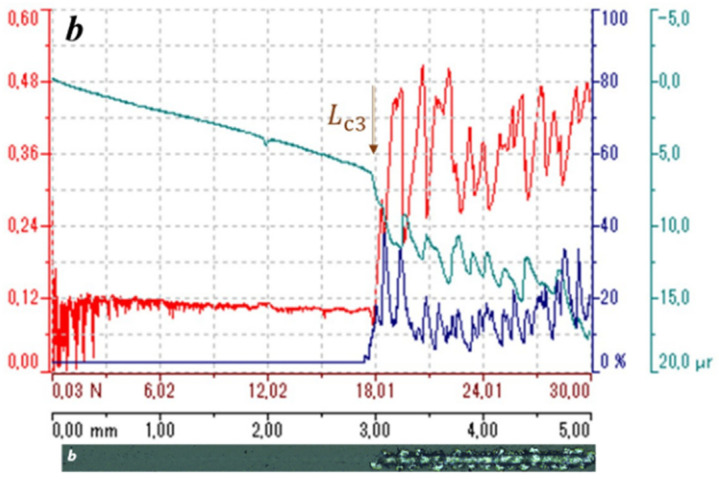
The friction coefficient μ (red lines), intensity of acoustic emission (blue), and indenter’s contact depth *h* (cyan) under progressive loading in 5 mm long scratch on the surface of BAM-coated R6M5 steel. Here, (**a**) and (**b**) frames correspond, respectively, to (**a**) and (**b**) images of the two scratches in Figure 8. The vertical arrows mark the positions of the critical load *L*_c3_.

The friction coefficient μ, intensity of acoustic emission, and indenter’s contact depth *h*, as shown in Figure 9, were recorded in BAM film under progressive loading *L* = 0–30 N. As seen, a linear increase in the applied load above 17–19 N led to instant and complete BAM coating failure, accompanied by chips and peeling. The adhesive strength of the BAM coating onto the R6M5 steel was about 18 N on average, and the friction coefficient before peeling was μ = 0.07. What is great is that BAM/R6M5 demonstrates so low friction in dry conditions without any lubricant applied. For example, a specially made solid-lubricant Ti_3_C_2_T*_x_*-graphene-oxide coating was sprayed onto a pre-heated knife 52100-steel substrate to achieve a substantial reduction in friction down to μ = 0.065 [31].

## 5. Work of BAM-Coating Adhesion onto R6M5 Steel

To characterize the film–substrate interfacial adhesion strength, some numerical criterion for film removal needed to be introduced. Laugier [32], and later Park and Kwon [33], suggested to associate it with the total deformation energy stored inside the coating layer as follows:

(2) $$W=t_{f}\frac{E_{f}}{2}\left(\varepsilon_{xx}^{2}+\varepsilon_{yy}^{2}\right),$$

where $t_{f}$ is the film layer thickness. The directions of the *x* and *y* axes are parallel and perpendicular, respectively, to the direction of the sliding indenter on the specimen’s surface. To calculate the strain components $\varepsilon_{ik}$ in the film, we used the formulae for the stress tensor $\sigma_{ik}$ derived by Hamilton and Goodman [34,35] (see also for corrections in ref. [36,37]):

(3) $$\begin{array}{c} \sigma_{xx}=\frac{L}{2\pi r^{2}}\left[1-2\nu_{s}-\eta \frac{3\pi }{8}\left(4+\nu_{s}\right)\mu \right],\\ \sigma_{yy}=-\frac{L}{2\pi r^{2}}\left[1-2\nu_{s}+\eta \frac{9\pi \nu_{s}}{8}\mu \right]. \end{array}$$

Here, in square brackets, the first terms are the radially symmetrical compressive and tensile elastic stress components, whereas the second term stand for the friction force-generated stress. Coefficients η=+1 and η=−1 correspond, respectively, to the leading and rear edges of the contact circle region with radius *r*. As a result, the strain in the coating can be expressed as follows:

(4) $$\begin{array}{c} \varepsilon_{xx}=\frac{1}{E_{f}}\left(\sigma_{xx}-\nu_{s}\sigma_{yy}\right)=\frac{L\left(1+\nu_{s}\right)}{2\pi r^{2}E_{f}}\left[1-2\nu_{s}-\eta \frac{3\pi }{8}\left(4-{3\nu }_{s}\right)\mu \right],\\ \varepsilon_{yy}=\frac{1}{E_{f}}\left(\sigma_{yy}-\nu_{s}\sigma_{xx}\right)=-\frac{L\left(1+\nu_{s}\right)}{2\pi r^{2}E_{f}}\left[1-2\nu_{s}-\eta \frac{3\pi \nu_{s}}{8}\mu \right]. \end{array}$$

Until the film adheres to the substrate, the contact radius *r* can be defined with an elastic Hertzian [38,39] formula as follows:

(5) $${r_{c}}^{3}=\frac{3}{4}L_{c}R\left(\frac{1-\nu_{s}^{2}}{E_{s}}+\frac{1-\nu_{f}^{2}}{E_{f}}\right)$$

Substituting in Equations (2)–(5), the radius of indenter *R =* 200 μm, film thickness $t_{BAM}$ = 3 μm, Young’s moduli $E_{R6M5}$ = 180 GPa and $E_{BAM}$ = 330 GPa, Poisson’s coefficients $\nu_{R6M5}$ = 0.295 and $\nu_{BAM}$ = 0.25, and friction coefficient μ = 0.07 before the coating fractures at a critical load of *L*_c3_ = 18 N, we obtained results for the contact radius *r*_c_ = 28 μm and the work of adhesion *W* = 63.6 J/m^2^.

It should be noted that the contributions of the friction force in Equations (3) and (4) are small due to a very low friction coefficient μ in comparison with the radial elastic stress produced by the indenter. This explains the circular shape of the very first and subsequent cracks that appeared in the tracks of the BAM coating in Figure 8.

## 6. Conclusions

High target sputtering RF power and short target-to-substrate distance guaranteed the fabrication of hard and highly adhesive AlMgB_14_ coatings onto industrial cutting tools. The micro- and nanohardness tests, evaluation of the abrasion and friction properties, and estimation of the interfacial adhesion strength characterized some of the prospects for the industrial use of BAM coatings.

R6M5 steel drills and WC-Co cemented carbide inserts became more than, respectively, three and two times harder after BAM coating. Great enhancement of the tool’s resistance to plastic deformation was the most impressive effect of BAM utilization. The BAM coating doubled the elastic recovery ratio *R*_e_. In the BAM-coated R6M5 samples, *R*_e_ reached as high as 75%, while the corresponding parameter *H*^3^/E*^2^ increased by a factor of ten. The doubled high elastic strain-to-failure ratio *H*/E* = 0.13 indicates BAM/R6M5 steel’s ability to elastically recover from deformation when the applied stress exceeds its elastic limit, thus decreasing abrasive exertion of the surface asperities. This property combined with high hardness, strength and durability, chemical inertness and unique lubricity (friction coefficient μ = 0.07) are key factors that promise great potential of magnetron-sputtered AlMgB_14_ coatings for novel applications in numerous industrial directions, such as cutting, punching and forming, molding, and die castling. The exploration of the high-temperature oxidation resistance of BAM coatings remains an important future task.

## Figures and Tables

**Table 1:** Results of the nanoindentation of the uncoated and BAM-coated WC-Co inserts.

Characteristics	BAM/WC-Co		WC-Co	
Loading force *L* (mN)	15	30	15	30
Maximum contact depth *h* (μm)	0.22	0.33	0.25	0.41
Hardness *H* (GPa)	23 ± 4	22 ± 4	15 ± 3	12 ± 3
Elastic modulus *E* (GPa)	320 ± 50	330 ± 60	600 ± 60	530 ± 80
Effective Young’s modulus E* (GPa)	340 ± 60	360 ± 70	620 ± 70	550 ± 90
Elastic recovery ratio *R*_e_ (%)	59 ± 5	53 ± 3	24 ± 6	26 ± 3
Elastic strain index *H*/E*	0.072	0.067	0.025	0.023
Resistance to plastic deformation ratio *H*^3^/E*^2^ (MPa)	119	98	9	6

**Table 2:** Results of the nanoindentation of the uncoated and BAM-coated AlTiN WC-Co inserts.

Characteristics	BAM/AlTiN/WC-Co		AlTiN/WC-Co	
Loading force *L* (mN)	15	30	15	30
Maximum contact depth *h* (μm)	0.21	0.32	0.25	0.40
Hardness *H* (GPa)	23 ± 3	21 ± 3	14 ± 3	14 ± 3
Elastic modulus *E* (GPa)	370 ± 60	380 ± 60	310 ± 80	300 ± 80
Effective Young’s modulus E* (GPa)	390 ± 60	400 ± 70	340 ± 80	320 ± 80
Elastic recovery ratio *R*_e_ (%)	55 ± 5	52 ± 6	41 ± 5	40 ± 3
Elastic strain index *H*/E*	0.062	0.055	0.045	0.047
Resistance to plastic deformation ratio *H*^3^/E*^2^ (MPa)	89	64	29	30

**Table 3:** Vickers microhardness HV0.2 for tungsten carbide turning inserts.

Sample	Microhardness HV0.2	
	(kgf/mm^2^)	(GPa)
Uncoated WC-Co	1531.74	15.02
BAM-coated WC-Co	3496.50	34.29
Uncoated AlTiN/WC-Co	2515.24	24.67
BAM-coated AlTiN/WC-Co	4589.24	45.01

**Table 4:** Results of the nanoindentation of the uncoated and BAM-coated R6M5 steel.

Characteristics	BAM/R6M5		R6M5	
Loading force *L* (mN)	15	30	15	30
Maximum contact depth *h* (μm)	0.17	0.27	0.28	0.44
Hardness *H* (GPa)	37 ± 3	40 ± 3	12.9 ± 0.7	12.3 ± 1.0
Elastic modulus *E* (GPa)	302 ± 17	305 ± 17	184 ± 12	170 ± 17
Effective Young’s modulus E* (GPa)	322 ± 18	325 ± 18	199 ± 13	184 ± 18
Elastic recovery ratio *R*_e_ (%)	75 ± 5	73 ± 4	38.2 ± 2.5	37.3 ± 1.9
Elastic strain index *H*/E*	0.123	0.131	0.070	0.072
Resistance to plastic deformation ratio *H*^3^/E*^2^ (MPa)	555	688	63	64

## Data Availability

The data presented in this study are available on request from the corresponding author.
